# Exercise-induced left bundle branch block: unmasking dynamic mitral regurgitation in ischaemic heart disease

**DOI:** 10.1093/ehjci/jeae011

**Published:** 2024-01-09

**Authors:** Gianluca Ricchetti, Eustachio Agricola, Massimo Slavich

**Affiliations:** Cardiovascular Imaging Unit, Cardio-Thoracic-Vascular Department, IRCCS San Raffaele Scientific Institute, Milan 20132, Italy; Cardiovascular Imaging Unit, Cardio-Thoracic-Vascular Department, IRCCS San Raffaele Scientific Institute, Milan 20132, Italy; Cardiovascular Imaging Unit, Cardio-Thoracic-Vascular Department, IRCCS San Raffaele Scientific Institute, Milan 20132, Italy; Department of Cardiology, IRCCS San Raffaele Scientific Institute, Milan 20132, Italy

A 70-year-old diabetic patient with a history of ischaemic heart disease underwent percutaneous coronary intervention with dual stent placement in the proximal left anterior coronary artery a decade ago. Experiencing exertional dyspnoea, the patient recently sought evaluation, leading to the performance of a stress echocardiography. The baseline electrocardiogram showed sinus rhythm with normal intraventricular conduction (*Panel A*). Echocardiogram revealed left ventricular hypertrophy with preserved systolic function, mild mitral regurgitation secondary to posterior leaflet prolapse, first-degree diastolic dysfunction, and normal pulmonary artery pressure (*Panel B*; Supplementary data online, *Video S1*). Global longitudinal strain was normal (*Panel C*). During stress, the patient developed a left bundle branch block (*Panel D*), associated with dyspnoea. Echocardiogram then showed extensive contraction dyssynchrony with preserved contractility of the anterior and inferior wall and severe mitral regurgitation (*Panel E*; Supplementary data online, *Video S2*). Strain pattern analysis confirmed mechanical dyssynchrony (*Panel F*). In the first minute of recovery, left bundle branch block resolved and mitral regurgitation downgraded to mild with resolution of symptoms. Coronary computed tomography showed no progression of coronary disease or intrastent restenosis.

This case highlights how isolated dyssynchrony can induce severe functional mitral regurgitation and emphasize the role of exercise tests in evaluating exertional symptoms, especially when out of proportion to overt structural abnormalities. In our patient, there was no ventricular dilatation or regional akinaesia, and left bundle branch block appeared only during exercise, associated with immediate evidence of clinically significant mitral regurgitation. Therefore, dynamic severe mitral regurgitation resulted from papillary muscle dyssynchrony, portraying dyspnoea as an angina equivalent.

**Figure:**
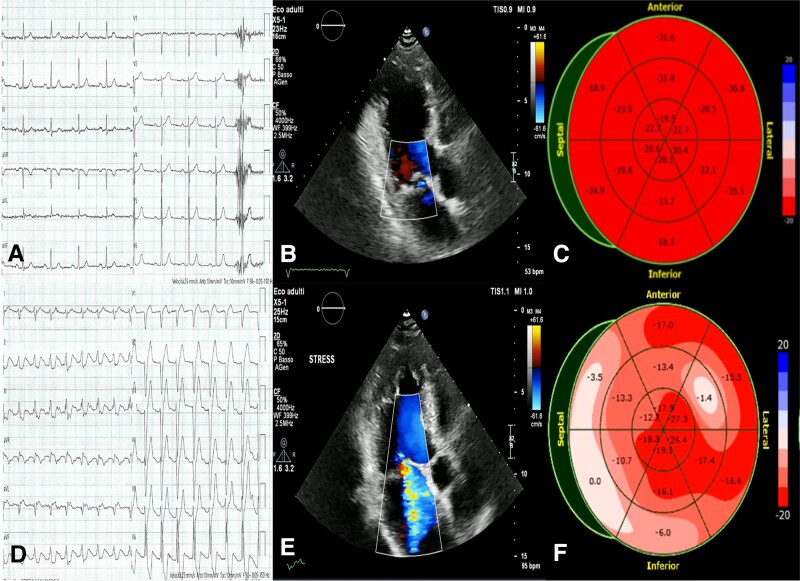


## Supplementary Material

jeae011_Supplementary_Data

## Data Availability

The data underlying this article are available in the article and in its online supplementary material.

